# Anti-VEGF intravitreal injections in the era of COVID-19: responding to different levels of epidemic pressure

**DOI:** 10.1007/s00417-021-05097-0

**Published:** 2021-02-02

**Authors:** Jean-François Korobelnik, Anat Loewenstein, Bora Eldem, Antonia M. Joussen, Adrian Koh, George N. Lambrou, Paolo Lanzetta, Xiaoxin Li, Monica Lövestam-Adrian, Rafael Navarro, Annabelle A. Okada, Ian Pearce, Francisco J. Rodríguez, David T. Wong, Lihteh Wu

**Affiliations:** 1 Service d’ophtalmologie, CHU Bordeaux, Bordeaux, France; 2 Inserm, Bordeaux Population Health Research Center, team LEHA, Université de Bordeaux, UMR 1219, F-33000 Bordeaux, France; 3 Division of Ophthalmology, Tel Aviv Medical Center, Sackler Faculty of Medicine, Tel Aviv University, Tel Aviv, Israel; 4 Department of Ophthalmology, Hacettepe University, Ankara, Turkey; 5 Charité–University Medicine Berlin, Berlin, Germany; 6 Eye and Retina Surgeons, Camden Medical Centre, Singapore, Singapore; 7 Global Medical Affairs Ophthalmology, Bayer, Basel, Switzerland; 8 Department of Medicine–Ophthalmology, University of Udine, Udine, Italy; 9 Department of Ophthalmology, Ospedale Santa Maria della Misericordia, Azienda Sanitaria Universitaria Friuli Centrale (ASUFC), Udine, Italy; 10 Istituto Europeo di Microchirurgia Oculare, IEMO, Udine, Italy; 11 Eye Center and Eye Institute, Peking University People’s Hospital, Beijing, China; 12 Department of Ophthalmology, Lund University Hospital, Lund, Sweden; 13 Instituto Microcirugia Ocular, Barcelona, Spain; 14 Department of Ophthalmology, Kyorin University School of Medicine, Tokyo, Japan; 15 Royal Liverpool University Hospital, Liverpool, UK; 16 Fundación Oftalmologia Nacional, Escuela de Medicina y Ciencias de la Salud, Universidad del Rosario, Bogotá, Colombia; 17 Unity Health Toronto–St. Michael’s Hospital, University of Toronto, Toronto, Ontario Canada; 18 Macula, Vitreous and Retina Associates of Costa Rica, San José, Costa Rica

**Keywords:** Retinal disease, Ophthalmology, COVID-19, Coronavirus, Recommendations, Vision Academy

## Abstract

**Purpose:**

Following the first wave of the COVID-19 pandemic in early 2020, the easing of strict measures to reduce its spread has led to a resurgence of cases in many countries at both the national and local level. This article addresses how guidance for ophthalmologists on managing patients with retinal disease receiving intravitreal injections of anti-vascular endothelial growth factor (VEGF) during the pandemic should be adapted to the local epidemic pressure, with more or less stringent measures implemented according to the ebb and flow of the pandemic.

**Methods:**

The Vision Academy’s membership of international retinal disease experts analyzed guidance for anti-VEGF intravitreal injections during the COVID-19 pandemic and graded the recommendations according to three levels of increasing epidemic pressure. The revised recommendations were discussed, refined, and voted on by the 14-member Vision Academy Steering Committee for consensus.

**Results:**

Protocols to minimize the exposure of patients and healthcare staff to COVID-19, including use of personal protective equipment, physical distancing, and hygiene measures, should be routinely implemented and intensified according to local infection rates and pressure on the hospital/clinic or healthcare system. In areas with many COVID-19-positive clusters, additional measures including pre-screening of patients, postponement of non-urgent appointments, and simplification of complex intravitreal anti-VEGF regimens should be considered. Treatment prioritization for those at greatest risk of irreversible vision loss should be implemented in areas where COVID-19 cases are increasing exponentially and healthcare resources are strained.

**Conclusion:**

Consistency in monitoring of local infection rates and adjustment of clinical practice accordingly will be required as we move forward through the COVID-19 era. Ophthalmologists must continue to carefully weigh the risk–benefits to minimize the exposure of patients and healthcare staff to COVID-19, ensure that patients receive sight-saving treatment, and avoid the potential long-term impact of prolonged treatment postponement.

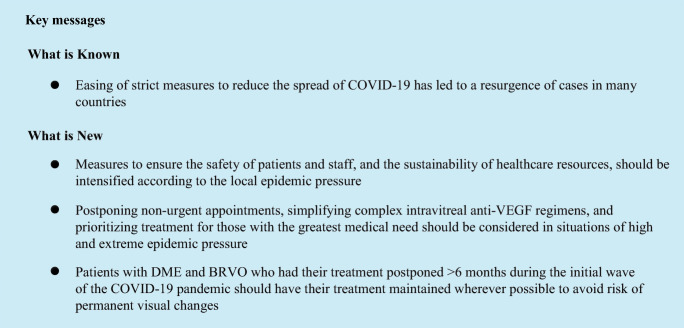

## Introduction

Following the rapid spread of the novel coronavirus SARS-CoV-2 across the world in early 2020, the ophthalmic community had to quickly adjust clinical practice in response to high infection rates, mounting pressure on healthcare systems, and implementation of restrictions or “lockdowns” that precluded many patients from attending appointments.

As we navigate through the current phase of the pandemic, where infection rates are once again accelerating in many countries and vary largely between regions, ophthalmologists must be prepared to respond quickly to the changing epidemic pressure in their local area to ensure that patients receive sight-saving ophthalmic care, while still ensuring the safety of patients and staff.

The Vision Academy previously published guidance for managing patients receiving anti-vascular endothelial growth factor (VEGF) injections during the acute phase of the COVID-19 pandemic [1]. As we adjust to the “new normal” of ophthalmic care in the era of COVID-19, we consider how this guidance should be implemented according to the local epidemic pressure. This article provides practical guidance for the management of patients receiving anti-VEGF injections while the threat of COVID-19 remains, and describes how measures should be escalated when infection rates rise and healthcare resources become stretched, to ensure prioritization of treatment for those with the greatest medical need. Conversely, this article also indicates how measures can be de-escalated when the epidemic pressure decreases.

## Methods

The Vision Academy is an international group of more than 90 retinal physicians who work together to share existing skills and knowledge, and provide collective recommendations on clinical challenges in areas where there is a lack of conclusive evidence in the literature [2].

Vision Academy guidance for anti-VEGF intravitreal injections during the COVID-19 pandemic was first published online in April 2020 [1], during the first “wave” of the pandemic. This guidance was reviewed during the Vision Academy Annual Meeting in August 2020, where members were asked to validate and decide which recommendations should be implemented at three levels of local epidemic pressure. Following contributions from the membership, the revised recommendations were analyzed, refined, and voted on by the 14-member Vision Academy Steering Committee for consensus.

## Guiding principles

Ensuring the safety of healthcare staff and patients should be a key consideration in all decision-making, and practices should be reviewed regularly to account for changing local epidemic pressure. Vigilance in identifying suspect cases of COVID-19 remains essential, with symptoms including dry cough, fever, and fatigue, or less commonly, loss of taste or smell, headache, muscle pain, sore throat, conjunctivitis, dyspnea, nasal congestion, skin rash, or diarrhea [3]. Patients receiving intravitreal injections of anti-VEGF are often elderly and/or diabetic, both of which are characteristics associated with a high risk of COVID-19 complications and hospitalization [4, 5]. While it is important to minimize the exposure of vulnerable patients to avoidable risk, prevention of irreversible vision loss through continuation of care should be practiced wherever possible.

Although our previous guidance [1] discussed the potential for short-term deprioritization of certain cases of diabetic macular edema (DME) and branch retinal vein occlusion (BRVO) due to a reduced likelihood for irreversible vision loss [6, 7], it is important to consider that many patients with DME and BRVO will have already had their treatment postponed during the initial wave of the COVID-19 pandemic, and it is possible that further deferral of treatment may lead to permanent visual changes.

Overall, patients receiving intravitreal injections require clear communication and advice to ensure they feel supported and reassured that their vision remains a key priority. Extensive considerations can be found in previously published guidance [1]. A summary of the recommendations is shown in Table 1.

**Table 1:** Guidance for anti-VEGF intravitreal injections in retinal disease patients according to local COVID-19 epidemic pressure [1]

Low epidemic pressure situations^a^	
General considerations	
• Regularly monitor medical/healthcare staff for signs and symptoms of infection	
• Provide staff with regular training on use of PPE and other safety practices	
• Consistently implement and follow personal, facility, and instrument hygiene/disinfection rules	
Prioritizing patients according to medical need	
• Discuss treatment prioritization with the patient, taking into account the legal/regulatory environment, status of the epidemic, and the capacity to reschedule postponed procedures	
• If necessary, prioritize treatment visits over monitoring visits o Inform patients on how to self-monitor their vision/implement the use of home monitoring technologies, if possible	
• Defer appointments of COVID-19-positive/suspect patients, except for cases requiring emergency intervention to prevent imminent danger of severe vision loss	
• Prior to the appointment, inform patients about the safety and hygiene measures in place • Provide a “Dear Patient” letter that reiterates the importance of attending appointments and offers advice on what to do should they be unable to attend [8]	
• Provide patient support via an emergency contact number manned by a senior ophthalmologist	
Reducing exposure during the patient visit	
• Ensure wearing of face masks at all times (patients and staff) [9–11] o An N95 or FFP2 mask is preferred or a surgical mask where these are not available	
• Ensure good ventilation in all rooms [12]	
• Limit exposure in waiting rooms by use of masks, 1m or 2 m physical distancing, spacing out appointments, allowing only one accompanying adult if necessary, and promoting queuing outside the waiting room	
Reducing exposure during the patient examination	
• Wear PPE for patients who are COVID-19-positive/suspect, or for all patients, as directed by local authorities	
• Keep examinations as brief as possible and consider implementing physical distancing measures between patients and staff	
• Thoroughly disinfect hands and equipment, including keyboards, between patients	
• Affix large plastic/plexiglass shields to slit lamps and OCT	
• To reduce risk of contamination, tape the upper edges of the face mask during intravitreal injection procedures	
• For COVID-19-positive/suspect patients, emergency surgery/intervention should take place in a facility with appropriate safety measures and PPE in place	
High epidemic pressure situations^b^, in addition to the above recommendations	
Prioritizing patients according to medical need	
• Pre-screen patients by phone to identify symptomatic/suspected COVID-19-positive patients (or relatives/caregivers) • Prioritize and maintain treatment schedules in patients with nAMD (particularly if they are in the first 2 years of treatment), new patients with significant vision loss, neovascular glaucoma, and monocular or quasi-monocular patients (only one eye > 20/40)	
• Consider postponement of appointments for non-monocular patients, except patients with significant vision loss from recent DME, proliferative diabetic retinopathy, acute-phase RVO, and ischemic RVO who should not be postponed	
• Avoid prolonged treatment postponement (> 4–6 months) and reassess the situation regularly (within 2–3 months)	
• Patients with DME and BRVO who already had their treatment postponed > 6 months during the initial wave of the COVID-19 pandemic should have their treatment maintained	
Reducing exposure during the patient examination	
• Limit the use of OCT examinations and special instruments unless they are critical to decision-making	
Treatment regimen considerations	
• Avoid treatment regimens and regimen changes that require frequent monitoring to adjust dosing intervals	
• Avoid switching treatment regimen unless there is a clear lack of response	
• Avoid changing treatment intervals in patients with nAMD who are responding to a fixed-dose regimen	
• Consider reverting to the last effective treatment interval and use this for fixed dosing in patients with AMD receiving variable-interval treatment regimens o Reassure patients that fixed-dose anti-VEGF regimens are an effective way of delivering treatment [7, 13, 14]	
• Maintain the loading phase schedule and select longer-acting therapies for new patients	
• Only consider reimplantation of a dexamethasone implant in patients with DME/RVO if they are responding well and have a history of normal intraocular pressure under such treatment	
• Consider panretinal photocoagulation instead of intravitreal anti-VEGF for patients with severe PDR	
Extreme epidemic pressure situations^c^, in addition to all the above recommendations	
Prioritizing patients according to medical need	
• Postpone non-urgent appointments where there is capacity to reschedule within ≤ 4–6 months	
• Prioritize and maintain treatment schedules in patients with nAMD (particularly if they are in the first 2 years of treatment), new patients with significant vision loss, neovascular glaucoma, and monocular or quasi-monocular patients (only one eye > 20/40)	
• Consider referral to a non-hospital-based clinic or ambulatory surgical center	
• Consider using telemedicine consultations for patient triaging and to monitor those whose in-person appointments have been postponed o It may be acceptable in the short term (≤ 4–6 months) to monitor the disease on function only	
• Consider offering home care for patients unable to attend in-person appointments (e.g., under lockdown)	
Reducing exposure during the patient examination	
• Avoid thorough visual acuity testing of every patient o A simple self-performed test such as a near-reading chart may be sufficient to flag an important visual change requiring further investigation	

^a^*R*_*t*_ significantly < 1 without herd immunity through mass vaccination; some physical distancing measures are likely to be implemented. These recommendations are also valid in situations with a higher alert level

^b^*R*_*t*_ ~ 1 and/or many clusters of COVID-19-positive people are present in the community but there is no strain on hospital resources. These recommendations are also valid in situations with a higher alert level

^c^*R*_*t*_ significantly > 1 and hospital resources are under significant pressure; lockdown measures may be in place. These recommendations are only valid in this alert level

*AMD* age-related macular degeneration, *BRVO* branch retinal vein occlusion, *DME* diabetic macular edema, *nAMD* neovascular age-related macular degeneration, *OCT* optical coherence tomography, *PDR* proliferative diabetic retinopathy, *PPE* personal protective equipment, *RVO* retinal vein occlusion, *VEGF* vascular endothelial growth factor

## Low epidemic pressure situations

The effective reproduction number, *R*_*t*_, is the expected number of new infections generated at time *t* by each infectious case, in a population where some individuals may no longer be susceptible [15, 16]. *R*_*t*_ has been widely used to monitor the spread of COVID-19, with achieving *R*_*t*_ < 1 being a key goal to prevent the exponential spread of infection [16–19]. When considering the following recommendations, low epidemic pressure indicates situations where *R*_*t*_ is significantly < 1 but herd immunity through mass vaccination has not yet been achieved. In such situations, some physical distancing measures are likely to be in place, but these would constitute the “new normal” until a suitable vaccine or treatment becomes available. Recommendations for situations of low epidemic pressure are also valid in situations with a higher alert level.

### General considerations

COVID-19 remains a threat even in areas where the infection rate is low, and vigilance must be practiced irrespective of the current situation to help prevent escalation of the infection rate. It is essential that safety and hygiene practices are implemented consistently throughout the patient journey to prevent the cycle of transmission (Table 1) [20].

### Prioritizing patients according to medical need

Even in situations of low epidemic pressure, ophthalmology clinics may be unable to run at full capacity due to physical distancing measures. If prioritization of patients is required, steps should be taken to ensure the patient is fully informed and that legal, regulatory, and future capacity considerations are appropriately assessed (Table 1). If necessary, treatment visits should be prioritized over monitoring visits, with self-monitoring procedures implemented where possible (Table 1) [21].

Postponing appointments for patients with confirmed or suspected COVID-19 is strongly recommended in all cases, with the exception of emergency intervention to prevent severe vision loss.

Measures to triage and support patients, and inform them of important safety practices such as use of masks and physical distancing [9–11] have been discussed previously [1]. In addition to these measures, it may be beneficial to provide a “Dear Patient” letter to all patients that reiterates the importance of attending appointments and offers advice on what to do should they be unable to attend [8].

### Reducing exposure during the patient visit

Specific considerations to reduce the exposure of patients and staff to COVID-19, including use of personal protective equipment (PPE), physical distancing, and other measures to reduce exposure in waiting rooms, have previously been described at length [1] and are summarized in Table 1. Importantly, use of masks and physical distancing are strongly encouraged due to their association with a reduced risk of infection in those exposed to COVID-19-positive individuals [9]. Risk reduction appears to be strongest with N95 or equivalent masks; both N95 and surgical masks may offer better protection from infection than single-layer masks. Physical distancing of at least 1 m is associated with a significant reduction in risk of infection, which may be further reduced by distancing by 2 m, and should be implemented wherever feasible [9].

### Reducing exposure during the patient examination

In addition to stringent hygiene measures, use of PPE, and physical distancing between staff and patients where appropriate, patient examinations should be kept as brief as possible (Table 1). Use of plastic/plexiglass shields affixed to slit lamps and optical coherence tomography may offer further protection during these examinations, in addition to the use of masks. Given the potential risk of contamination, taping the upper edges of the face mask during intravitreal injection procedures should be considered to prevent air jets from radiating towards the eyes [22].

## High epidemic pressure situations

High epidemic pressure indicates situations where the *R*_t_ is ~ 1 and/or there are many clusters of COVID-19-positive people present in the community. The following considerations are applicable for situations where the risk of contracting COVID-19 is high but hospital resources are not yet strained. Recommendations for situations of high epidemic pressure are also valid in situations with a higher alert level, where the *R*_*t*_ rises above 1 and hospital resources are strained, resulting in the need for patient prioritization.

### Prioritizing patients according to medical need

In situations where there is a high presence of COVID-19 in the community, additional safety measures should be implemented, such as pre-screening patients by phone to determine whether they have symptoms consistent with COVID-19 or have been recently exposed to the virus (Table 1).

High epidemic pressure may result in the need for postponement of non-urgent appointments, guidance for which has been previously published [1]. Patients with certain conditions may be at greater risk of vision deterioration or permanent vision loss with treatment delay and should have their treatment maintained (Table 1). Previous studies have suggested that, in the short term, DME and BRVO cases may be less likely to suffer irreversible vision loss [6, 7], which may make certain patients suitable candidates for treatment delay. However, as these patients may have already had their treatment postponed > 6 months during the initial wave of the COVID-19 pandemic, further deferral could lead to permanent visual changes and therefore these patients should have their treatment maintained wherever possible.

### Reducing exposure during the patient examination

In addition to the recommended guidance for low epidemic pressure situations (Table 1), it may be appropriate during high epidemic pressure to limit the use of optical coherence tomography examinations and special instruments (Table 1), unless they are deemed critical for the management of a particular patient.

### Treatment regimen considerations

Simplification of treatment regimens and avoidance of regimen changes that require frequent monitoring are recommended as additional measures to reduce exposure and free up healthcare resources during periods of high epidemic pressure. Guidance on suggested approaches to minimize the need for monitoring is provided in Table 1.

Due to the increased likelihood that patients will be unable to attend regular appointments during the pandemic, panretinal photocoagulation may be a preferable treatment choice for patients with severe proliferative diabetic retinopathy to reduce the potential risk of developing tractional retinal detachment.

It is important to reassure patients who are switched to a fixed-dose regimen as a result of the pandemic of the validity and efficacy of using this approach to deliver their anti-VEGF therapy [7, 13, 14].

## Extreme epidemic pressure situations

Extreme epidemic pressure indicates situations where the *R*_*t*_ is significantly > 1 and hospital resources are likely to be under significant pressure. In such circumstances, where the spread of COVID-19 is accelerating rapidly, it is likely that lockdown measures are implemented within the local area or country. The following recommendations are only for consideration in instances of extreme epidemic pressure and where the risk–benefits have been carefully weighed.

### Prioritizing patients according to medical need

Where the risk of infection is high and hospital resources are strained, consider postponing non-urgent appointments where there is capacity to reschedule within ≤ 4–6 months (Table 1).

Additional measures, including implementation of telemedicine consultations, referral of patients to non-hospital-based settings, or offering home care, should be considered where possible to reduce footfall at hospitals and limit the risk of exposure of vulnerable patients to COVID-19. Telemedicine consultations are encouraged for patients who have been deprioritized to enable visual function monitoring until they are able to attend an in-person appointment, which should ideally be within ≤ 4–6 months (Table 1).

### Reducing exposure during the patient examination

In situations of extreme epidemic pressure, it may be acceptable to avoid full visual acuity testing of every patient in order to reduce the examination time and any potential exposure. Use of a near-reading chart or performance of a brief visual acuity test may be sufficient to flag any visual changes that require further investigation (Table 1).

## Conclusion

Management strategies for ophthalmic care of patients with retinal disease while COVID-19 remains a threat should be reassessed at regular intervals and adjusted in response to local infection rates and the availability of healthcare resources. The long-term impact of the delays or cancellations of ophthalmology appointments during the initial wave of the COVID-19 pandemic is still to be determined. As long as local infection rates remain low, ophthalmologists should aim to practice at as close to normal operating levels as possible, to limit the risk of irreversible vision loss while ensuring that adequate safety protocols, including PPE and physical distancing, are in place.

In areas where there are many COVID-19-positive clusters or where the number of cases is increasing exponentially, measures to ensure the safety of patients and staff, and the sustainability of healthcare resources, should be intensified as appropriate. In such situations, complex intravitreal anti-VEGF treatment regimens requiring frequent monitoring and dose adjustment should be simplified, and treatment should be prioritized for those at greatest risk of irreversible vision loss.
